# AVID: An integrative framework for discovering functional relationships among proteins

**DOI:** 10.1186/1471-2105-6-136

**Published:** 2005-06-01

**Authors:** Taijiao Jiang, Amy E Keating

**Affiliations:** 1 Department of Biology, Massachusetts Institute of Technology, Cambridge, USA

## Abstract

**Background:**

Determining the functions of uncharacterized proteins is one of the most pressing problems in the post-genomic era. Large scale protein-protein interaction assays, global mRNA expression analyses and systematic protein localization studies provide experimental information that can be used for this purpose. The data from such experiments contain many false positives and false negatives, but can be processed using computational methods to provide reliable information about protein-protein relationships and protein function. An outstanding and important goal is to predict detailed functional annotation for all uncharacterized proteins that is reliable enough to effectively guide experiments.

**Results:**

We present AVID, a computational method that uses a multi-stage learning framework to integrate experimental results with sequence information, generating networks reflecting functional similarities among proteins. We illustrate use of the networks by making predictions of detailed Gene Ontology (GO) annotations in three categories: molecular function, biological process, and cellular component. Applied to the yeast *Saccharomyces cerevisiae*, AVID provides 37,451 pair-wise functional linkages between 4,191 proteins. These relationships are ~65–78% accurate, as assessed by cross-validation testing. Assignments of highly detailed functional descriptors to proteins, based on the networks, are estimated to be ~67% accurate for GO categories describing molecular function and cellular component and ~52% accurate for terms describing biological process. The predictions cover 1,490 proteins with no previous annotation in GO and also assign more detailed functions to many proteins annotated only with less descriptive terms. Predictions made by AVID are largely distinct from those made by other methods. Out of 37,451 predicted pair-wise relationships, the greatest number shared in common with another method is 3,413.

**Conclusion:**

AVID provides three networks reflecting functional associations among proteins. We use these networks to generate new, highly detailed functional predictions for roughly half of the yeast proteome that are reliable enough to drive targeted experimental investigations. The predictions suggest many specific, testable hypotheses. All of the data are available as downloadable files as well as through an interactive website at . Thus, AVID will be a valuable resource for experimental biologists.

## Background

High-throughput technologies, including genome sequencing, expression profiling, and large-scale interaction and localization assays, have provided a wealth of data about proteins and their properties, particularly for the model organism *Saccharomyces cerevisiae *[1-5]. The process of inferring functional information from these data is not straightforward. The data are not of uniformly high quality and must be weighted in an appropriate way [6-8]. Computational methods such as machine learning hold promise for this task, but they require a clear definition of "protein function". Gene Ontology (GO) has emerged as a unifying framework that makes it possible to carry out computational function annotation [9].

GO uses expert curation to systematically describe the role of proteins in the cell. Three hierarchical ontologies are used: molecular function (MF) describes the specific molecular task performed by a protein, biological process (BP) describes the broader biological activity a protein participates in, and cellular component (CC) describes the subcellular location or complex where a protein is found. The deeper an annotation is in one of the GO hierarchies, the more informative and specific it is. For example, at a low level the yeast protein Mcm2 is annotated with "catalytic activity" as a molecular function, but at the most detailed level of the MF hierarchy its description is "ATP-dependent DNA helicase activity". Similarly, Mcm2's biological process of "cell growth and/or maintenance" is refined to "DNA unwinding", and the general descriptor "nucleus" is refined as "pre-replicative complex" at the most descriptive level of the CC classification. An important goal is to provide the most detailed possible annotations for all proteins. Currently, however, only ~60% of the *S. cerevisiae *proteome has annotation at the most descriptive level of at least one of the MF, BP or CC classifications.

Many groups have explored computational analysis as a way to expand functional assignments. For example, the genomic context of a gene can reveal functional relationships, especially in prokaryotes, as can patterns of co-evolution [10-12]. Several methods have been proposed for using experimental protein-protein interaction networks to assign functional descriptors to unknown proteins on the basis of their interaction partners [13-17]. Other researchers have developed methods for combining multiple sources of data [11,12,18,19]. Troyanskaya *et al*. used a Bayesian approach to increase the accuracy of functional predictions of GO BP terms [20], and Jansen *et al*. predicted new members of protein complexes in this way [21,22]. Tanay *et al*. (2004) integrated several sources of experimental data to generate statistically significant protein "modules" and used the modules to assign GO BP terms to 874 uncharacterized yeast proteins [23]. Most recently, Lee *et al*. used a Bayesian framework to build networks reflecting functional relationships between 4,677 yeast proteins [24].

We present a method called AVID (Annotation Via Integration of Data) for predicting functional relationships among proteins. AVID integrates the results of high-throughput experiments, and incorporates sequence data, to build unified, high-confidence networks in which proteins are connected if they are likely to share a common annotation. We illustrate one use of these networks by treating functional annotation as a classification problem and assigning GO terms to individual proteins based on their neighbors in the networks. AVID is distinct from previous computational function prediction methods in several ways that will make it a useful tool for experimental biologists. First, AVID predicts functional annotation in all three GO categories: MF, BP and CC. This provides a more complete view of an uncharacterized protein's possible role in the cell than any single term alone. Second, the functional terms predicted by AVID are very detailed. We adopted only the most specific terms in GO as a list of possible annotations and refer to these as *AVID GO terms*. AVID GO terms have no functional subcategories. There are 841 AVID GO terms for MF, 602 for BP and 192 for CC that are used for yeast. Such terms are considerably more useful than general ones, and they are also harder to predict. Third, by considering five different types of input data, AVID achieves good coverage; we report here predictions of new GO terms for about half of the yeast genome. Fourth, AVID is reliable. The functional networks generated are 65–78% accurate and annotation of proteins is 52–67% accurate. The trade-off obtained between coverage and accuracy is superior to that obtained using a naïve Bayesian framework. Finally, AVID predicts relationships among proteins that are largely distinct from those that have been suggested by other computational methods [12,13,20,21,24-26].

Here, we describe three stages used in AVID to construct functional correlation networks and a fourth stage that is used to assign specific functions to individual proteins. We report the estimated accuracy of AVID at different stages using known data. Then we describe the results of applying AVID to the entire yeast proteome to generate new GO annotations.

## Results and discussion

### Description and performance of the four stages of AVID

**Figure 1:**
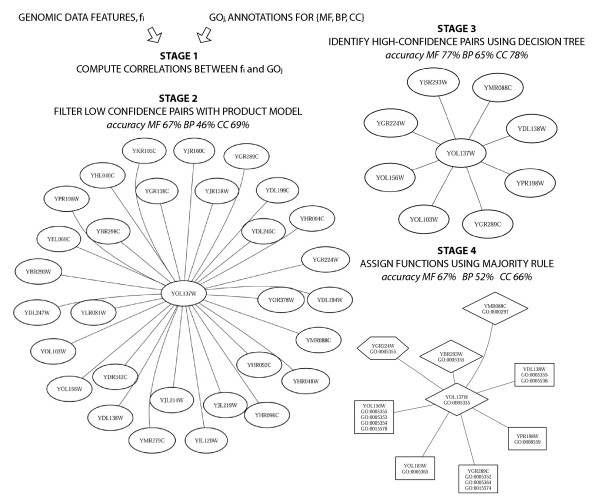
**Overview of AVID, using prediction of the molecular function of YOL137W as an example**. Initially, YOL137W is treated as potentially functionally related to 6,448 other yeast proteins (not shown). Stage 2 prunes this to 30 putatively similar proteins, and the stage 3 decision tree further reduces this list to eight high-confidence neighbors. See additional file 2 for a description of the decision tree. In stage 4, five of the eight neighbors of YOL137W have existing AVID GO annotation (boxed) and "vote" to assign the GO term GO:0005355. After stage 4, proteins with known annotation in the figure are boxed, those with novel predictions are shown in diamonds and the function of YGR224W, in a hexagon, is a refined prediction. The estimated accuracy of predicted functional similarities (in stages 2 and 3) and annotations (in stage 4) are given in italics (see Methods). MF: molecular function; BP: biological process; CC: cellular component. Rosette figures generated using Graphviz [46].

Figure 1 outlines the multi-stage method and its application to predicting the MF of unannotated protein YOL137W. AVID can be regarded as a filtering process. Initially, all proteins are considered as potentially functionally related. Stages 2 and 3 remove lower confidence associations. The links remaining after stage 3 form networks that contain a wealth of information about functional similarity. These networks are the primary output of AVID, and in stage 4 they are used to assign specific functional terms to individual proteins.

In stage 1, diverse features, such as the presence or absence of sequence similarity, or the observation of a protein-protein interaction, are considered as potential indicators of whether two proteins share an AVID GO term (Table 1 and additional file 1). For each type of evidence, i, and each GO category, j = {MF, BP, CC}, we define a correlation coefficient P_ij_^AVID1 ^to describe how well the evidence predicts functional similarity. For all three ontologies, MF, BP and CC, there is a weak positive correlation between the experimental observation of an interaction (by yeast 2-hybrid or by co-purification) and similarity of annotation. Sequence similarity is correlated with MF and BP, but less so with CC, consistent with the expectation that evolutionarily related proteins frequently have related functions but act in diverse parts of the cell. The cellular localization data of Huh et al. [1] correlate positively but very weakly with CC. This is because GO cellular components, at the most detailed level, are protein complexes that are much more descriptive than this experimental localization data. AVID GO terms in the CC classification should often, in fact, be regarded as descriptions of protein interaction rather than cellular localization. Thus it is not surprising that, e.g., two proteins sharing the location "nucleus" have a low probability of participating in the same complex (and thus sharing an AVID GO CC term). Gene co-expression profiles correlate with all three GO functional types; the higher the Pearson correlation between two expression profiles is, the more likely the two proteins share a GO term of any kind. Most of the correlations between data and function are very weak; none of the correlation coefficients are greater than 35%. Nevertheless, differences between the (+) and (-) data sets (see Table 1) make these sources valuable for inferring functional similarity.

**Figure 2:**
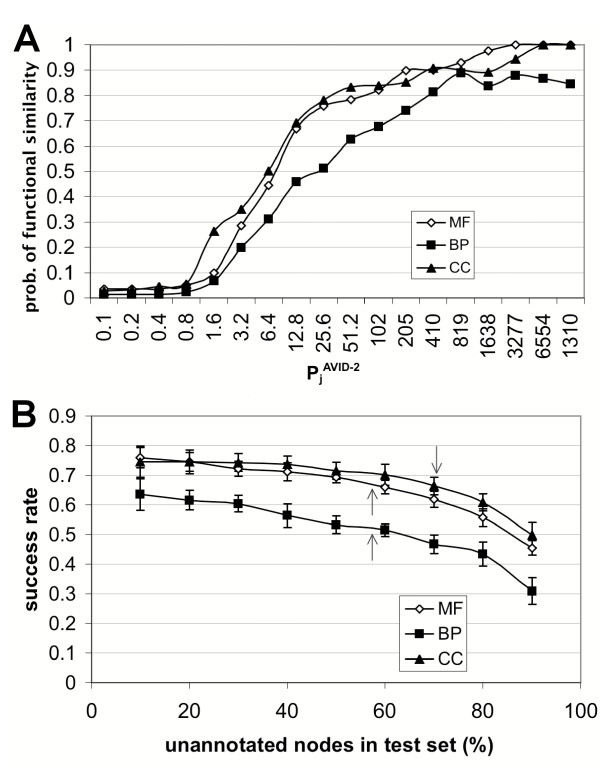
**Evaluation of stages 2 and 4 using known proteins**. (**A**) To evaluate the performance of a simple product model in stage 2, P_j_^AVID2 ^was calculated for each pair of proteins in an annotated reference set. The plot shows the fraction of protein pairs at different P_j_^AVID2 ^cutoffs that share an AVID GO term. Pairs with P_j_^AVID2 ^< 12.8 were not considered in stages 3 or 4. (**B**) A varying percentage of reference proteins was omitted from the entire training procedure and used as a test set. Functions for these proteins were predicted in stage 4, and the plot shows the success rate for correctly predicting at least one existing GO term at the highest level of annotation. The arrows indicate the expected performance for predicting new functions based on the current status of annotation of the yeast genome. Error bars show the standard deviation from 100 random cross-validation trials. MF (diamonds), BP (squares) and CC (triangles).

Stage 2 is a filter that combines data sources and removes protein pairs that lack sufficient evidence of functional relatedness. For each pair of proteins, a P_j_^AVID2 ^value is defined as the product of the normalized conditional probabilities P_ij_^AVID1 ^from all sources of evidence in stage 1. Tests using known proteins show that pairs with a P_j_^AVID2 ^value greater than 12.8 have more than 66.8%, 45.8% or 69.3% probability of MF, BP or CC relatedness (Figure 2A). Pairs with P_j_^AVID2 ^< 12.8 are not considered further. Good coverage is preserved using this cutoff: 60.8%, 74.0% or 79.0% of proteins in test sets with existing MF, BP or CC annotation are retained by the filter.

To improve accuracy while still making predictions for a large number of proteins, a machine learning scheme (a decision tree) is employed in stage 3 [27]. The tree takes the stage 1 conditional probabilities as input, and returns a binary decision about whether two proteins are likely to share a function. The entire process used to predict the similarity of three pairs of proteins is illustrated in detail in additional file 2, which includes diagrams of the decision trees for MF, BP and CC.

Stages 1, 2 and 3 result in the construction of a reliable protein correlation network for each of the GO functional types. The three networks relate proteins likely to share a similar function or be part of the same protein complex. Ten-fold cross-validation testing using proteins with existing GO annotation showed that 77% of MF, 65% of BP and 78% of CC pair-wise relations predicted in stage 3 were correct. Furthermore, 1,432 proteins (55.4%), 1,122 proteins (48.6%) and 974 proteins (72.3%) for MF, BP and CC, respectively, were retained in pairs judged to have functional similarity after stage 3. Alternative machine-learning strategies were tested but did not show any improvement in performance. All of the stages employed to construct the networks are important. In particular, a decision tree applied to unfiltered data was not as effective, resulting in 4–21% reduced coverage and 2–3% reduced accuracy for BP and CC, and ~10% reduced accuracy for MF, compared to the full 3-stage process.

In stage 4, a "majority rule" algorithm is used to annotate uncharacterized proteins based on the correlation networks. AVID often predicts several GO terms for a protein. The average number of functions predicted (compared to the number of existing annotations in GO, in parentheses) is 2.19 (1.45) for MF, 1.95 (1.07) for BP, and 1.87 (1.14) for CC. In testing, if any of the AVID predictions were identical to an existing GO annotation the prediction was counted as correct. If one term was correctly predicted, all terms were correctly predicted 85% of the time for MF, 74% of the time for BP and 83% of the time for CC.

Figure 2B shows that the success rates for predicting AVID GO terms in stage 4 depend on the fraction of proteins treated as unknown in the networks during testing. If only 10% of proteins have known function, the success rate for the remaining 90% (the test set) is only 30–50%. However, if functions are known for 90%, the remaining 10% of proteins can be annotated with 65–75% accuracy. Whereas the assessment data in Figure 2B are based on reference sets of annotated proteins, divided into training and testing sets, the actual fraction of yeast proteins currently unannotated at the AVID GO level is 56.4%, 56.5% and 70.3% for MF, BP and CC, respectively. Thus we can use Figure 2B to estimate that MF, BP and CC prediction success, when applied to the entire proteome, will be ~67%, ~52% and ~66%, respectively. As more proteins are annotated, predictive accuracy for unknown proteins will improve further.

As mentioned above, AVID predicts 1.5 to 1.8 times as many AVID GO terms per protein as already exist in GO. This is expected, because current annotation is incomplete. In tests on annotated proteins, the percentage of functional terms predicted by AVID that are already included in GO is 46%, 33% and 47% for MF, BP and CC, respectively. We recover 63% (MF), 44% (BP) and 60% (CC) of previously annotated AVID GO terms.

### New networks including unannotated *S. cerevisiae *proteins

We applied AVID to the entire yeast proteome, generating three new networks that include proteins for which detailed AVID GO annotation is not yet available. These predicted networks have similar connectivity to the networks generated from existing GO data for testing. The average numbers of edges per node in the testing networks were 10.2, 8.2, and 17.2 for MF, BP and CC, respectively, whereas in the prediction networks these averages were 13.4, 8.7 and 12.5. The distribution of connectivities in the different networks are provided in additional file 3. Using the predicted networks, we made two types of functional predictions. All predictions are available in additional files 4 and 5.

#### Refined predictions

First, we predicted a more detailed function or location for proteins previously characterized only at less descriptive levels in GO, we refer to these as *refined predictions*. We evaluated whether refined predictions are consistent with existing coarser annotations at GO levels 2, 3 and 4 for MF, BP and CC, respectively. An AVID GO prediction was judged consistent with a coarser one if the less descriptive term is its ancestor in the GO hierarchy. There are 17 functional categories in MF level 2, 24 functional categories in BP level 3 and 40 functional categories in the CC level 4, so this comparison is a non-trivial test. Because the definition of "level" can be ambiguous, the categories used are listed in additional file 6.

Table 2 summarizes the results; refined predictions are 75 – 87% consistent with existing coarser annotations. Note that these coarser annotations were not used by AVID in any way when predicting specific terms. This indicates that new AVID GO terms, when traced back to MF level 2, BP level 3 or CC level 4, are 75–87% accurate. This estimate is higher than the accuracy for predicting AVID GO terms themselves because it is easier to assign more general functions correctly. For cases in which a refined AVID prediction is *not *a subcategory of existing GO annotation, we identified many cases where the prediction is, nevertheless, biologically relevant. In a trivial example, AVID assigns YOR244W (Esa1) a MF of "chromatin binding". Our formal accounting treated this as incorrect in testing because "chromatin binding" is not a sub-category of the existing GO term for Esa1, "histone acetyltransferase activity". However, Esa1 catalyzes acetylation of chromatin substrates [28], so the disagreement in this test is merely an artefact of the structure of GO. This suggests that refined AVID GO predictions are more consistent with existing biological knowledge than the estimate of 75–87%.

#### Novel predictions

**Figure 3:**
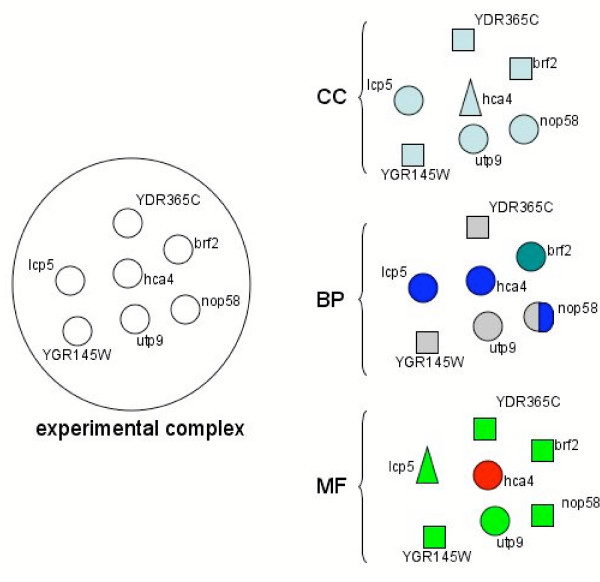
**GO and AVID annotations of proteins localized to an experimentally identified complex**. The complex shown at left was identified using co-purification/mass spectrometry by Hazbun et al. [30]. On the right, proteins with known AVID GO terms are shown as circles; proteins with refined AVID predictions are shown as triangles, proteins with novel AVID predictions are shown as squares. Colors represent AVID GO terms as follows: light blue – small nucleolar ribonucleoprotein complex; grey – processing of 20S pre-rRNA; dark blue – 35S primary transcript processing; dark green – ER to Golgi transport; light green – snoRNA binding; red – ATP dependent RNA helicase activity. This complex is predicted to have a role in RNA transcript processing. The refined and novel functional predictions agree with previous annotations and with each other, increasing confidence that these are meaningful assignments.

In a second type of prediction, we assigned one or more AVID GO terms to proteins without any existing annotation in GO at any level, we refer to these as *novel predictions*. Note that these are novel in the sense that they annotate proteins not previously included in GO. Other sources of evidence regarding function may exist, e.g. in the literature or at the Saccharomyces Genome Database (SGD) [29]. We made novel MF predictions for 950 proteins, novel BP predictions for 504 proteins and novel CC predictions for 907 proteins (Table 2). The refined and novel predictions for MF, BP and CC together cover 51% of the yeast proteome and, when combined with existing annotations, provide AVID GO descriptors for ~80% of yeast proteins. Cross-validation testing indicated that these predictions are ~52–67% accurate (previous section). The accuracy of specific novel predictions is hard to evaluate systematically, but we assessed the plausibility of a subset of both our refined and novel predictions using the experimental data of Hazbun et al. [30] (Figure 3), which were not included in the version of GO used to develop our method. In this work, 100 uncharacterized open reading frames were labelled with a tandem affinity purification tag. Mass spectrometry was used to identify proteins that form a complex with the gene product of interest. Overlap with the high-throughput affinity purification data used as input to AVID was very low (7%). For each novel annotation predicted by AVID for a protein localized to a complex by Hazbun et al., we classified it as *GO-consistent *if another member of the complex had the same annotation in GO, and as *AVID-consistent *if another member of the complex had the same annotation predicted by AVID. We found that ~46% of MF, ~16% of BP and ~27% of CC predictions were GO-consistent; ~73% (43 out of 59) of MF, 63% (27 out of 44) of BP and 71% (45 out of 62) of CC predictions were GO- or AVID-consistent. Among the annotations not formally classified as GO- or AVID-consistent some are nevertheless clearly relevant. For example, AVID assigns "mitotic chromosome segregation" to YNL313C. In the Hazbun experiments, YNL313C co-purified with Tub3p, and Tub3p is annotated in GO with the very similar term "homologous chromosome segregation". Thus, AVID predicted the functional similarity of YNL313C and Tub3, and this was supported later by the co-purification of these proteins. Notably, the AVID prediction of similarity of YNL313C and Tub3 did not come directly from experimental evidence; there is no direct edge between these two proteins in any of the AVID networks.

AVID predictions of MF, BP and CC were made using different weighting of the input data, different functional categories and different stage 3 correlation networks. For 85 proteins, AVID provided novel predictions in all three of these categories. In many examples, the three novel predictions are related and consistent (additional file 7). Here we list several examples from these 85 proteins where experimental data, or descriptions in SGD, support our predictions. First, YOR179C (Syc1) was assigned by AVID a BP of "mRNA polyadenylation", a MF of "cleavage/polyadenylation specificity factor activity" and CCs of "mRNA cleavage factor complex" and "cleavage and polyadenylation specificity factor complex". GO annotation added after our predictions were completed assigned YOR179C "mRNA cleavage and polyadenylation specificity factor complex" as a cellular component, based on the experiments of Nedea *et al*. [31]. In another example, AVID assigned "tRNA-intron endonuclease activity" (MF), "tRNA splicing" (BP), and "tRNA-intron endonuclease complex" (CC), to YLR375W (see additional file 2). SGD lists the description "involved in pre-tRNA splicing and in uptake of branched-chain amino acids" for YLR375W, although this information is not included in GO or supported by literature references at SGD [29]. In a third example, AVID assigned YEL018W (Eaf5), YER092W (Ies5) and YDL002C (Nhp10) to "histone acetylation" (BP), "chromatin binding" (MF), and "nucleosome remodelling complex" (CC); SGD reports for Eaf5 the description "subunit of the NuA4 acetyltransferase complex", and for Ies5 "protein that associates with the INO80 chromatin remodelling complex under low-salt conditions". The involvement of Nhp10 in chromatin remodelling is also supported by Shen *et al*. [32]. Similar consistency among MF, BP and CC predictions supports the annotation of YBR043C, YIL121W (Qdr2) and YOL137W (Bsc6) as plasma membrane-associated proteins involved in glucose transport and/or galactose metabolism and the assignment of YER084W, YGR017W, YLR154C (Rnh203) and YPL014W as being related to (possibly regulating) protein kinase CK2 activity. Many other suggestive examples are available in additional file 7.

We have constructed an interactive web server that allows users to individually trace the data that contributed to any prediction [33]. For example, to understand the origins of the MF prediction made for YOL137W, shown in Figure 1, a user can look up the identities and functions of all neighbors of this protein in the stage 3 networks. For each neighbor, we provide information about what experimental or sequence data was used to establish the relationship, as well as the stage 1 weight assigned to that data source, and an additional measure of confidence from the decision tree processing (see Methods). We also give its status as "known", "refined" or "novel". This makes it possible to establish, for example, that YOL137W was assigned GO term 0005355 ("glucose transporter activity") because the majority rule vote by neighbors of known function was won by YOL156W and YDL138W, which share GO term 0005355. The association of these proteins with YOL137W was determined primarily from sequence similarity and mRNA co-expression. Users can also see, however, that YOL137W is predicted to share functional similarity with other proteins, e.g. YOL103W (GO:0005365, "myo-inositol transporter activity"), also on the basis of sequence similarity and mRNA co-expression. This demonstrates how examining AVID network relationships can provide a broader picture than the stage 4-assigned terms alone.

### Comparison to other methods

A variety of methods have been proposed in the literature for the computational annotation of protein function, but these are difficult to compare given the lack of adequate "gold standard" data sets for universal testing. There are further obstacles to rigorous comparison. First, groups formulate the function prediction problem differently. For example, our use of highly specific GO terms is distinct from the generally broad (and widely varying) functional classifications used by others. Second, methods use different sources of evidence and training sets. Finally, various methods provide different forms of output, ranging from sets of pair-wise relationships between proteins to functional modules to specific functional annotations.

**Figure 4:**
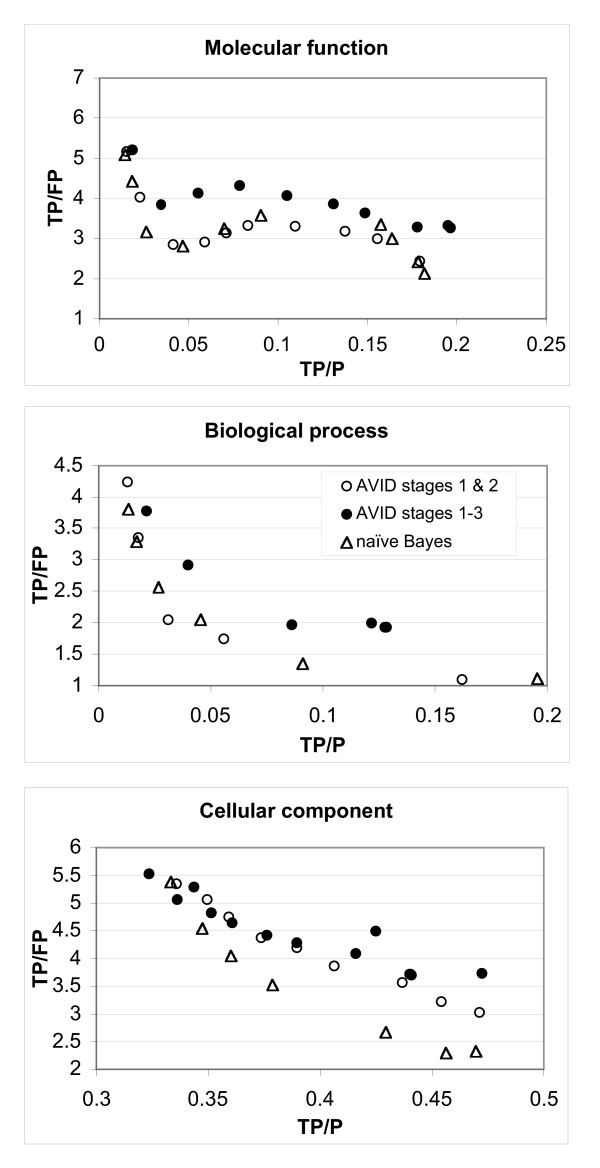
**Comparison of AVID with a naïve Bayesian network**. The performance of a naïve Bayesian net, as described by Jansen et al. [21], is compared to that of AVID, using the same input data and same measures of performance. The plots show the ratio of true positives to false positives (TP/FP) vs. coverage (TP/P). The Bayesian net results are in open triangles, AVID stages 1 and 2 in open circles and AVID stages 1, 2 and 3 in closed circles. The performance of AVID is notably superior at higher coverage. At low coverage, all methods can achieve high accuracy.

Bayesian frameworks are popular for data integration problems, and have been applied to the prediction of protein functional similarity and protein-protein interactions [20,21,24]. To compare the AVID framework rigorously with a naïve Bayesian net, we implemented the method of Jansen et al. [21] and applied it to our set of reference proteins. As shown in Figure 4, AVID stages 1 and 2 perform comparably to this formalism for MF and BP, although they out-perform it for CC. With the addition of the decision tree in stage 4, however, the trade-off between accuracy and coverage improves significantly for AVID. Whereas both methods are good at making high-confidence predictions at low coverage, the AVID framework maintains much better true positive to false positive ratios than the naïve Bayes net at higher coverage.

Although implementing and comparing a large number of other approaches for the same data is beyond the scope of this work, we can compare overall results obtained by different groups. This turns out to be interesting and surprising. For six published methods that generate pair-wise relationships among yeast proteins, we compare the coverage and overlap of the predicted associations in Table 3. We compare methods at roughly comparable levels of accuracy to that of AVID (~70%), using estimates of the original authors. AVID predicts 37,451 relationships among 4,191 proteins. Lee et al. [24], referred to here and in Table 3 as "MARCOTTE") also obtain very high coverage: 33,919 high-confidence associations among 4,677 proteins. STRING further predicts 23,345 functionally related pairs. However, the largest overlap between any two methods in Table 3 is only 9,873 pair-wise associations predicted by both MARCOTTE and STRING [12,26]. Both of these methods use genomic context as an important predictive element. AVID does not consider genomic context and shares only 3,413 predictions with STRING. These make up 9% of the total AVID predictions, and no other method shows greater overlap. Out of 37,451 high-confidence associations predicted by AVID and 33,919 by MARCOTTE, only 3,020 of these are in common. In light of the fact that all methods show incomplete coverage and imperfect accuracy, the distinct predictions made by different methods are a significant advantage because they provide alternatives that can profitably be considered by experimentalists.

## Conclusion

Computational annotation of the proteome has a critical role to play in post-genomic analysis. Although hypotheses about function can often be reached by carefully reading the literature and critically examining high-throughput data, computation can speed and assist this process. Further, computational methods can help discriminate reliable data amidst false positives and negatives. As a tool for this purpose, AVID notably provides functional descriptors at a high level of detail. The strategy of predicting MF, BP and CC terms also provides a more comprehensive description of protein function than many alternative approaches. Finally, AVID performs better than simple naïve Bayesian integration, and the predictions of AVID are largely distinct from those that have been made by other methods.

The stage 3 networks generated by AVID are very accurate (65–78%) and are useful in the absence of stage 4. The specific predictions made in stage 4 are accurate enough to be of practical utility, but they do have limitations. The imperfect majority rule algorithm will sometimes select one function over others that may be equally relevant. Further, because we consider only the most detailed GO categories in training and prediction, some predictions will be incorrect because they are overly specific, even when they correctly reflect the general cellular role of a protein. For these reasons, consideration of the entire stage 3 functional networks is likely to be most useful to experimental biologists. Other algorithms for assigning function based on the AVID networks may give better performance. This is an active area of research [13-16].

AVID can be used to assign new proteins to existing GO functional categories. The catalogue provided by GO is incomplete, however. Accurate descriptors have not yet been defined for all possible functions, processes and compartments. Because of this, proteins with new functions will not be successfully assigned by AVID. Within the limitations imposed by GO, however, performance on novel proteins may be better than estimated by our testing. When assessing the performance of stage 4, we treated known proteins as unknown. This reduced the size of the training set for stage 3 to less than half of that available when making new predictions; this decreases predictive accuracy. Furthermore, most proteins have more than one function, and many are found in more than one cellular compartment or complex. When assessing AVID stage 3, predicted functional similarities among test proteins that are not yet annotated in GO are counted as wrong and thus reduce the estimated success rate, even though many are likely to be correct.

Algorithmic function prediction can be approached from different perspectives, and it will be important for computational biologists to explore various formulations of the problem as well as solutions to it. Ultimately, the value of any approach will be justified through the cumulative success of experiments that it inspires. Our functional networks provide numerous candidate proteins for involvement in important biological processes. Biologists who consult AVID as part of their work are likely to find new predictions of function for their genes of interest that are accurate enough to guide experimental characterization. Thus, AVID is sure to provide a useful resource for the yeast community.

## Methods

### Data sources

To establish sequence similarity, 6,449 protein sequences from the yeast proteome were downloaded from MIPS [34]. Each sequence in turn was used as a PSI-BLAST query against the entire yeast proteome. Proteins with an E-value less than 0.001 after three iterations of PSI-BLAST were defined as similar to the query sequence [35]. A total of 66,833 similar pairs involving 3,631 proteins were identified in this manner.

A list of high-throughput yeast two-hybrid interactions was obtained from MIPS (file PPI_120803.tab) that included 6,620 pairs among 3,579 proteins (not including 214 self interactions) detected by the high-throughput yeast two-hybrid assays of Uetz and Ito [4,5,36]. Small-scale yeast two-hybrid experiments were not included.

Protein complex file complex052102.tab was downloaded from MIPS. Among these proteins, 67,569 pair-wise relationships were defined between 2,696 proteins reported to occur in the same complex [2,3]. Within a complex, every protein was assigned an interaction with all others.

We used the cellular localization data of Huh *et al*. [1,37]. A link was assigned to two proteins if they were reported in the same cellular compartment, without considering ~270 proteins with ambiguous localization. This led to the construction of 975,891 pairs among 3,883 proteins. In Table 1 this data set is called "UCSF localization".

The data GDS124 was downloaded from NCBI Gene Expression Omnibus [38]. We used cdc15 block-release time course mRNA expression from the yeast cell cycle. These data consist of 24 time points taken during the course of almost three full cell cycles. We computed the Pearson correlation for each of 19,734,903 pairs among 6,283 proteins.

Yeast protein annotations and hierarchical terms for biological processes, molecular functions and cellular components were downloaded from GO [39]. We only considered annotation categories that do not have any sub-categories. We call these AVID GO terms. A pairing link is assigned to two proteins if they share an AVID GO term.

Data from small-scale experiments were not used. We found that small-scale experiments are already largely captured by existing GO annotation. Furthermore, as implemented, the results we obtain with AVID reflect what is likely to be possible in other organisms, where high-throughput data sets will soon far outnumber small-scale experiments.

### Stages of AVID

The following stages were carried out separately for MF, BP and CC.

#### AVID stage 1 – correlation analysis

We considered five features, f_i_, that characterize pairs of proteins. Four of these features are either present (f_i_^+^) or absent (f_i_^-^) for a protein pair: co-localization, two-hybrid interaction, co-occurrence in a complex and sequence similarity. A fifth feature, mRNA expression profile correlation, was described by the Pearson correlation coefficient R. R values were binned into 19 intervals. We considered three GO categories, GO_j _(one of MF, BP, CC). Conditional probabilities were defined using only the set of proteins, s_ij_, that had records for both f_i _and GO_j_. GO_ij_^+ ^is the set of all protein pairs among s_ij _that share an AVID GO_j _term. For each feature, i, and GO category, j, we computed the conditional probability that a pair of proteins that share feature f_i _also share some AVID GO_j _annotation term: P_ij_^AVID1^= P(common GO term|common data feature) = (pairs in GO_ij_^+ ^with f_i_)/(pairs with f_i_). Because protein pairs lacking a relationship (e.g. an interaction) are frequently not reported, the number of such negative pairs was defined as {(possible pairs among p) – (pairs observed to have the corresponding positive feature)}. The correlation coefficients P_ij _^AVID1 ^were normalized by a factor α = 0.01/ [(pairs in s_ij _and in GO_ij_^+^)/(pairs among s_ij_)] to account for the different sizes and compositional biases of the sets s_ij_. The value 0.01 is a reference constant used in place of P_ij_^AVID1 ^for protein pairs without records in f_i_; it is approximately the probability that two randomly chosen proteins will share an AVID GO term. The analysis is insensitive to the value chosen for this constant.

#### AVID stage 2 – combining data to build a network of correlations

Each pair of proteins in the positive and negative reference sets was described by a set of five P_ij_^AVID1 ^terms for each of MF, BP and CC; the reference value 0.01 was used when feature data was missing (see above). In stage 2 we computed P_j_^AVID2 ^= Π_i_100•P_ij_^AVID1^. Pairs of proteins with P_j_^AVID2 ^< 12.8 for MF, BP and CC were not considered further. This cutoff was chosen to achieve a good compromise between accuracy and coverage, which can not be simultaneously optimized. Coincidentally, the 12.8 value was reasonable for all three GO categories.

#### AVID stage 3 – decision tree

In stage 3, the P_ij_^AVID1 ^values from stage 1 for protein pairs that passed the stage 2 filter were used as the input to a decision tree that retuned a binary decision about the presence or absence of functional similarity [27]. Decision trees provide a supervised machine learning scheme for classification and can analyze hierarchical complex relationships. The idea is to recursively subdivide a training set of examples into homogeneous groups, using discriminating attributes. The attribute selection criteria are based on a measure of informational entropy. At each decision point, an attribute is chosen so as to result in the best discrimination of the data into classes. After training, a set of complex rules is represented as a tree structure where non-terminal nodes represent tests on one or more attributes and terminal nodes reflect decision outcomes. We adopted java source code from Weka [40] for the implementation of the decision tree. J48 is an extension to the C4.5 algorithm by Quinlan [41]. It uses a recursive "divide and conquer" strategy to generate a decision tree from the training data. The input training data consist of a list of examples with attribute values and a class label (in our case it is YES or NO to represent correlation or not). Following Zhang et al. [19], we computed a measure of confidence in different paths through the final trees used for making new predictions. This measure is the probability that the decisions made to reach a particular terminal node correctly classified reference data; it is defined for each terminal node and is assigned to each protein pair partitioned to that node. These values are available at the AVID web site.

#### AVID stage 4 – assigning functions based on correlations

Pairs predicted to be functionally related by the decision tree in stage 3 comprise three correlation networks (one each for MF, BP and CC). In stage 4 these networks are used to classify unknown proteins based on their relationships to categorized neighbors. We assigned functions to unclassified proteins on the basis of the most common function(s) present among their annotated neighbors (the "majority rule" approach) [17]. Wherever possible, functions were assigned based only on GO-annotated proteins. When this was impossible (e.g. no neighbors had known functions), subsequent rounds of majority rule were used to assign functions on the basis of predicted annotations for neighbors. We also tested several iterative methods, such as those discussed by Vazquez et al. [14], but did not find any improvement in performance.

### Cross-validation testing

The performance of the AVID framework was evaluated using cross-validation testing by splitting the data into training and test sets prior to the stage 1 correlation analysis. We defined test sets in which increasing percentages (n) of the reference proteins were treated as unknown. The remaining 100-n% of proteins constituted the training set and were used in stages 1, 2 and 3 to generate a predictive model. This model was applied to the entire reference set to generate functional correlation networks. In these networks, the n% of proteins making up the test set remained unannotated. One or more functions were assigned to them using majority rule, and these predictions were compared to original terms assigned by GO. If at least one AVID GO term matched, the annotation of that protein was counted as correct. Accuracy was defined as the number of proteins with ≥ 1 correctly predicted AVID GO term divided by the number of proteins treated as unannotated. For each value of n, ranging from 10 to 90%, 100 random test sets were analyzed (for each of MF, BP and CC). The results reported are the average of all trials, with error bars in Figure 2b showing the standard deviations.

### Comparison with other methods

We compared AVID with a naïve Bayesian network approach used by Jansen et al. [21], which is described in detail in the supplementary materials of that reference. In this formalism, the probability of two proteins sharing functional annotation, given evidence sources f_1_...f_n_, is proportional to the likelihood ratio L: L(f_1_...f_n_) = P(f_1_...f_n_|functional similarity)/P(f_1_...f_n_|no functional similarity). Data features f_1_...f_n _are defined above in the section describing AVID stage 1. L can be computed from contingency tables relating these data features with pairs of reference proteins that are and are not functionally related. These tables are given in additional file 8. Figure 4 compares the performance of AVID stages 1 and 2, AVID stages 1–3 and the naïve Bayes approach by plotting the ratio of true positives to false positives (TP/FP) as a function of sensitivity (defined as TP/P) for reference data. We generated data at various values of TP/FP and TP/P by varying the values of L and the cutoff for P_j_^AVID2^.

In Table 3 we compare data from other studies with the content of the AVID functional correlation networks. The following datasets were downloaded from the indicated sources. The cutoff applied (if any) was chosen based on the original reference to generate "high-confidence" protein pairs. Where possible, as detailed below, the cutoff was chosen to result in roughly 70–80% accuracy, to match the estimated accuracy of the AVID pairs. The numbers reported for AVID include edges predicted for all three networks (MF, BP and CC); no protein pair was counted more than once.

1. **MARCOTTE**. "ConfidentNet", file 1099511s1_5.zip, from the supplementary materials of Lee et al. [24]; the top 34,000 pairs were used. These data are estimated by the authors to be as accurate as small-scale experiments; possibly greater than 70% accurate.

2. **STRING**. From the STRING database at [42], the file links_high_confidence_v5_1.txt [12,26]. This data set is reported to be ≥ 75% accurate.

3. **PIP_600**. From the work of Jansen et al. [21], file L_cut_PIP_600.tar from [43]. These predictions are theoretically estimated to be ~50% accurate.

4. **LIANG**. From the work of Samanta and Liang [13], all pairs with p < 10^-8 ^from [44]. These data are reported as ~70–75% accurate for predicting similarity in broad categories.

5. **MAGIC **From the data of Troyanskaya et al. [20], predictions.txt from [45]. We used two data sets, one with a probability of being functionally related of ≥ 50%, the other with ≥70%.

6. **SCHLITT**. File Schlitt-11114_supp_3.txt, with p ≤ 0.01, from the supplementary material of [25]. This p-value cutoff was used in the paper, but what accuracy it corresponds to isn't established.

## Authors' contributions

TJ and AEK conceived these studies and analyzed and interpreted the data and their presentation. TJ carried out all of the programming and computation. AK wrote the article, which was read and approved by TJ.

## Supplementary Material

Additional File 1 Correlation coefficients and supporting data in detail. Click here for file

Additional File 2 **Detailed description of the AVID process**. The first three stages of AVID are illustrated by tracing the prediction of functional relationships between (1) YOL137W and YDL138W, (2) YLR375W and YDR463W, and (3) YIR003W and YIL034C. The structures of the MF, BP and CC decision trees are included. Click here for file

Additional File 3 Connectivity plots comparing the testing and prediction networks. Click here for file

Additional File 4 AVID MF, BP and CC predictions – refined and novel. Click here for file

Additional File 5 Number of proteins with each AVID GO term – original and predicted. Click here for file

Additional File 6 MF level 2, BP level 3, CC level 4 categories. Click here for file

Additional File 7 Proteins with novel predictions in all three categories: MF, BP and CC Click here for file

Additional File 8 Contingency tables for the naïve Bayesian analysis Click here for file

## Figures and Tables

**Table 1:** Correlation between experimental or sequence-based measures of protein relatedness and GO annotation similarity.

**Data**^a^	**MF**^b^	**BP**^b^	**CC**^b^
UCSF localization (+)	0.031	0.029	0.058
UCSF localization (-)	0.007	0.007	0.004
yeast 2-hybrid (+)	0.120	0.167	0.254
yeast 2-hybrid (-)	0.010	0.010	0.010
MIPS complex (+)	0.122	0.116	0.263
MIPS complex (-)	0.007	0.008	0.002
sequence similarity (+)	0.187	0.344	0.078
sequence similarity (-)	0.009	0.007	0.010
Microarray^c^			
-1 < R ≤ -0.9	0.008	0.003	0.006
0.9 < R ≤ 1.0	0.086	0.118	0.125

^a^Except for the microarray data, each data source is divided into two sets: one contains protein pairs observed to share the feature described by the data (+), the other contains protein pairs that lack the feature or are not reported (-). ^b^The normalized conditional probabilities P_ij>_^AVID1 ^are defined in the Methods; they are the probability of sharing a common GO term given observation of a particular data feature. A perfect correlation would give a value of 1.0. ^c^R is the Pearson correlation coefficient for pairs of mRNA expression profiles, which were binned into 19 intervals. Only two intervals are shown here. See additional file 1 for further details.

**Table 2:** Summary of AVID prediction performance.

	**MF**	**BP**	**CC**
total predicted proteins	1852	1458	2304
proteins with novel predictions (no existing GO annotation)	950	540	907
proteins with refined predictions (existing GO annotation is less detailed)	902	918	1397
accuracy of stage 3 pair-wise similarities, from 10-fold cross validation	77%	65%	78%
frequency of unannotated proteins in the correlation networks	56.4%	56.5%	70.3%
estimated success rate for AVID GO term assignment	~67%	~52%	~66%
consistency of refined predictions with existing annotation	74.7%	80.3%	86.9%

**Table 3:** Overlap of pair-wise predictions of functional (or localization) similarity by different methods.

**METHODS**	**AVID**	**MARCOTTE**	**STRING**	**PIP_600**^a^	**LIANG**	**MAGIC**^b^	**MAGIC**^c^	**SCHLITT**
**AVID**	37451	3020	3413	785	2570	2740	49	9
**MARCOTTE**		33919	9873	3528	2454	1971	66	26
**STRING**			23245	3614	1740	1819	32	21
**PIP_600**^a^				9897	425	260	8	1
**LIANG**					7963	1647	41	6
**MAGIC**^b^						7922	397	7
**MAGIC**^c^							397	1
**SCHLITT**								526

a. This study was intended to predict pairs of proteins that co-localize to the same complex.

b. MAGIC pairs with p ≥ 0.5

c. MAGIC pairs with p ≥ 0.7
