# Supplementary material for: AVID: An integrative framework for discovering functional relationships among proteins
Source: BMC Bioinformatics. 2005 Jun 1;6:136. doi: 10.1186/1471-2105-6-136 (PMC1177925; doi:10.1186/1471-2105-6-136)
Supplement: Additional File 2 — Detailed description of the AVID process. The first three stages of AVID are illustrated by tracing the prediction of functional relationships between (1) YOL137W and YDL138W, (2) YLR375W and YDR463W, and (3) YIR003W and YIL034C. The structures of the MF, BP and CC decision trees are included. [file 1471-2105-6-136-S2.pdf]

Jiang & Keating
AVID: An integrative framework for discovering functional relationships among proteins
Additional file 2
This file contains three examples illustrating the process used to predict functional similarity. The structure of the decision trees used for MF, BP and CC predictions are also included here.

A. YOL137W and **YDL138W** (see Figure 1). YOL137W is predicted to have glucose transporter activity, based in part on its predicted similarity to YDL138W. The process of predicting the MF similarity of these two proteins illustrates that the decision is based primarily on sequence similarity. Note, however, that even when evidence is missing or negative it can influence the final network structure (as illustrated in the decision tree) and provide higher confidence in final predictions.

Stage 1 – Evidence (available at http://bmc-140.mit.edu/avid for all predictions)
UCSF localization
there is no data relating these proteins in the UCSF localization study, $P_{UCSF,MF}^{AVID1} = 0.01$ (default)
Yeast 2-hybrid
these proteins are included high-throughput yeast 2-hybrid, but are not observed to interact in any assay, $P_{Y2H,MF}^{AVID1} = 0.0099$
MIPS complex
these proteins are not part of a common complex described in MIPS, $P_{complex,MF}^{AVID1} = 0.01$ (default)
sequence similarity
there is a significant sequence similarity between these proteins, $P_{seq,MF}^{AVID1} = 0.187$
mRNA profile
there is mRNA expression profile data for these two proteins, but the correlation coefficient for the two mRNA profiles (R = 0.2865) correlates weakly with MF, $P_{mRNA,MF}^{AVID1}$ = 0.01 04

Stage 2 - Filtering
P_{MF}^{AVID2} for this pair is the product of 100 times the correlation coefficients determined in Stage 1, with a value of 1.0 (= 100 x 0.01) used when there is no data.
$P_{MF}^{AVID2} = 18.7$ is greater than 12.8, so this pair passes the Stage 2 filter.

Stage 3 - The MF Decision Tree
The $P_{i,j}^{AVID1}$ values are fed to a decision tree, and are used as the basis for a series of binary decisions about whether two proteins are functional related (YES = related; NO = not related). The path through the tree for YOL137W and YDL138W is underlined. The same tree structure is used for all MF decisions.

```
mips_complex <= 0.01
|   sequence_similarity    <= 0.01
|   |   yeast2hybrid <= 0.009877
|   |   |   mips_complex <= 0.007243: YES
|   |   |   mips_complex > 0.007243: NO
|   |   yeast2hybrid > 0.009877
|   |   |   mRNA_profile_R  <= 0.008011: YES
|   |   |   mRNA_profile_R  > 0.008011: NO
|   sequence_similarity    > 0.01
|   |   mRNA_profile_R  <= 0.016433
|   |   |   mips_complex <= 0.007243
|   |   |   |   mRNA_profile_R  <= 0.010425: NO
```

```
|   |   |   |   mRNA_profile_R  > 0.010425
|   |   |   |   |   yeast2hybrid <= 0.009877: NO
|   |   |   |   |   yeast2hybrid > 0.009877
|   |   |   |   |   |   mRNA_profile_R  <= 0.011242: NO
|   |   |   |   |   |   mRNA_profile_R  > 0.011242: YES
|   |   |   mips_complex > 0.007243
|   |   |   |   UCSFLocalization <= 0.01
|   |   |   |   |   yeast2hybrid <= 0.009877
|   |   |   |   |   |   mRNA_profile_R  <= 0.009376
|   |   |   |   |   |   |   mRNA_profile_R  <= 0.008011: YES
|   |   |   |   |   |   |   mRNA_profile_R  > 0.008011: NO
|   |   |   |   |   |   mRNA_profile_R  > 0.009376: YES
|   |   |   |   |   yeast2hybrid > 0.009877
|   |   |   |   |   |   mRNA_profile_R  <= 0.010008: NO (1000.0/408.0)
|   |   |   |   |   |   mRNA_profile_R  > 0.010008
|   |   |   |   |   |   |   UCSFLocalization <= 0.006564: NO
|   |   |   |   |   |   |   UCSFLocalization > 0.006564: YES
|   |   |   |   UCSFLocalization > 0.01: YES
|   |   mRNA_profile_R  > 0.016433
|   |   |   mRNA_profile_R  <= 0.02429
|   |   |   |   yeast2hybrid <= 0.009877
|   |   |   |   |   mips_complex <= 0.007243: NO
|   |   |   |   |   mips_complex > 0.007243
|   |   |   |   |   |   UCSFLocalization <= 0.01: YES
|   |   |   |   |   |   UCSFLocalization > 0.01: NO
|   |   |   |   yeast2hybrid > 0.009877: YES
|   |   |   mRNA_profile_R  > 0.02429
|   |   |   |   mRNA_profile_R  <= 0.038853
|   |   |   |   |   UCSFLocalization <= 0.006564: YES
|   |   |   |   |   UCSFLocalization > 0.006564
|   |   |   |   |   |   yeast2hybrid <= 0.009877
|   |   |   |   |   |   |   mips_complex <= 0.007243
|   |   |   |   |   |   |   |   UCSFLocalization <= 0.01: NO
|   |   |   |   |   |   |   |   UCSFLocalization > 0.01: YES
|   |   |   |   |   |   |   mips_complex > 0.007243: YES
|   |   |   |   |   |   yeast2hybrid > 0.009877: YES
|   |   |   |   mRNA_profile_R  > 0.038853: YES
mips_complex > 0.01
|   UCSFLocalization <= 0.01
|   |   sequence_similarity    <= 0.009188
|   |   |   yeast2hybrid <= 0.009877
|   |   |   |   mRNA_profile_R  <= 0.02429: NO
|   |   |   |   mRNA_profile_R  > 0.02429
|   |   |   |   |   UCSFLocalization <= 0.006564: NO
|   |   |   |   |   UCSFLocalization > 0.006564
|   |   |   |   |   |   mRNA_profile_R  <= 0.038853: YES
|   |   |   |   |   |   mRNA_profile_R  > 0.038853: NO
|   |   |   yeast2hybrid > 0.009877
|   |   |   |   UCSFLocalization <= 0.006564: NO
|   |   |   |   UCSFLocalization > 0.006564
|   |   |   |   |   yeast2hybrid <= 0.01
|   |   |   |   |   |   mRNA_profile_R  <= 0.016433: NO
|   |   |   |   |   |   mRNA_profile_R  > 0.016433: YES
|   |   |   |   |   yeast2hybrid > 0.01: YES
|   |   sequence_similarity    > 0.009188
|   |   |   UCSFLocalization <= 0.006564
|   |   |   |   sequence_similarity    <= 0.01
|   |   |   |   |   yeast2hybrid <= 0.009877
```

```
|   |   |   |   |   |   mRNA_profile_R  <= 0.016433: NO
|   |   |   |   |   |   mRNA_profile_R  > 0.016433: YES
|   |   |   |   |   yeast2hybrid > 0.009877
|   |   |   |   |   |   yeast2hybrid <= 0.01: NO
|   |   |   |   |   |   yeast2hybrid > 0.01
|   |   |   |   |   |   |   mRNA_profile_R  <= 0.009376: NO
|   |   |   |   |   |   |   mRNA_profile_R  > 0.009376: YES
|   |   |   |   sequence_similarity    > 0.01
|   |   |   |   |   mRNA_profile_R  <= 0.00889: NO
|   |   |   |   |   mRNA_profile_R  > 0.00889: YES
|   |   |   UCSFLocalization > 0.006564: YES
|   UCSFLocalization > 0.01: YES
```

B. YLR375W and YDR463W. YLR375W is predicted to be involved in tRNA splicing, based in part on its predicted similarity to YDR463W. The process of predicting the BP similarity of these two proteins illustrates that the decision is based on co-localization, sequence similarity and the fact that they have been identified as parts of a common complex. Note, however, that even when evidence is missing or negative it can influence the final network structure (as illustrated in the decision tree) and provide higher confidence in final predictions.
Note that SGD has reserved the name "STP" for YLR375W and includes in the description field "Involved in pre-tRNA splicing and in uptake of branched-chain amino acids", although this was not known to AVID (because the description is not included in GO).

Stage 1 – Evidence (available at http://bmc-140.mit.edu/avid for all predictions)
<u>UCSF localization</u>
these proteins are reported to co-localize in the UCSF localization study, $P_{UCSF,BP}^{AVID1} = 0.029$
<u>Yeast 2-hybrid</u>
these proteins are not both included in any high-throughput yeast two-hybrid study, $P_{Y2H,BP}^{AVID1} = 0.01$ (default)
<u>MIPS complex</u>
these proteins are part of a common complex described in MIPS, $P_{complex,BP}^{AVID1} = 0.116$
<u>sequence similarity</u>
there is a significant sequence similarity between these proteins, $P_{seq,BP}^{AVID1} = 0.344$
<u>mRNA profile</u>
there is mRNA expression profile data for these two proteins, but the correlation coefficient for the two mRNA profiles (R = 0.096) correlates weakly with MF, $P_{mRNA,BP}^{AVID1} = 0.0095$

Stage 2 - Filtering
P_{BP}^{AVID2} for this pair is the product of 100 times the correlation coefficients determined in Stage 1, with a value of 1.0 (= 100 x 0.01) used when there is no data.
$P_{BP}^{AVID2} = 1157.216$ is greater than 12.8, so this pair passes the Stage 2 filter.

Stage 3 - The BP Decision Tree
The $P_{i,j}^{AVID1}$ values are fed to a decision tree, and are used as the basis for a series of binary decisions about whether two proteins are functional related (YES = related; NO = not related). The path through the tree for YLR375W and YDR463W is underlined. The same tree structure is used for all BP decisions.

```
mips_complex <= 0.007956
|   UCSFLocalization <= 0.007428
```

```
|   |   microarray_GDS124_pairs <= 0.009008
|   |   |   yeast2hybrid <= 0.009812
|   |   |   |   microarray_GDS124_pairs <= 0.00768: NO
|   |   |   |   microarray_GDS124_pairs > 0.00768: YES
|   |   |   yeast2hybrid > 0.009812: YES
|   |   microarray_GDS124_pairs > 0.009008: NO
|   UCSFLocalization > 0.007428
|   |   yeast2hybrid <= 0.01
|   |   |   microarray_GDS124_pairs <= 0.009801: YES
|   |   |   microarray_GDS124_pairs > 0.009801
|   |   |   |   microarray_GDS124_pairs <= 0.01
|   |   |   |   |   UCSFLocalization <= 0.01: NO
|   |   |   |   |   UCSFLocalization > 0.01: YES
|   |   |   |   microarray_GDS124_pairs > 0.01
|   |   |   |   |   yeastProteome_paralogs <= 0.01: NO
|   |   |   |   |   yeastProteome_paralogs > 0.01: YES
|   |   yeast2hybrid > 0.01: NO
mips_complex > 0.007956
|   mips_complex <= 0.01
|   |   yeastProteome_paralogs <= 0.01: NO
|   |   yeastProteome_paralogs > 0.01
|   |   |   yeast2hybrid <= 0.009812
|   |   |   |   microarray_GDS124_pairs <= 0.006949: YES
|   |   |   |   microarray_GDS124_pairs > 0.006949
|   |   |   |   |   microarray_GDS124_pairs <= 0.01
|   |   |   |   |   |   UCSFLocalization <= 0.01
|   |   |   |   |   |   |   UCSFLocalization <= 0.007428: NO
|   |   |   |   |   |   |   UCSFLocalization > 0.007428: YES
|   |   |   |   |   |   UCSFLocalization > 0.01
|   |   |   |   |   |   |   microarray_GDS124_pairs <= 0.008145: NO
|   |   |   |   |   |   |   microarray_GDS124_pairs > 0.008145: YES
|   |   |   |   |   microarray_GDS124_pairs > 0.01
|   |   |   |   |   |   UCSFLocalization <= 0.01: NO
|   |   |   |   |   |   UCSFLocalization > 0.01
|   |   |   |   |   |   |   microarray_GDS124_pairs <= 0.01178
|   |   |   |   |   |   |   |   microarray_GDS124_pairs <= 0.010321: YES
|   |   |   |   |   |   |   |   microarray_GDS124_pairs > 0.010321: NO
|   |   |   |   |   |   |   microarray_GDS124_pairs > 0.01178: YES
|   |   |   yeast2hybrid > 0.009812
|   |   |   |   yeast2hybrid <= 0.01
|   |   |   |   |   microarray_GDS124_pairs <= 0.021518: NO
|   |   |   |   |   microarray_GDS124_pairs > 0.021518
|   |   |   |   |   |   UCSFLocalization <= 0.007428
|   |   |   |   |   |   |   microarray_GDS124_pairs <= 0.044563: NO
|   |   |   |   |   |   |   microarray_GDS124_pairs > 0.044563: YES
|   |   |   |   |   |   UCSFLocalization > 0.007428: YES
|   |   |   |   yeast2hybrid > 0.01
|   |   |   |   |   UCSFLocalization <= 0.007428: NO
|   |   |   |   |   UCSFLocalization > 0.007428: YES
|   mips_complex > 0.01
|   |   yeastProteome_paralogs <= 0.01
|   |   |   yeast2hybrid <= 0.01
|   |   |   |   UCSFLocalization <= 0.01: NO
|   |   |   |   UCSFLocalization > 0.01
|   |   |   |   |   yeastProteome_paralogs <= 0.007397
|   |   |   |   |   |   microarray_GDS124_pairs <= 0.013799: NO
|   |   |   |   |   |   microarray_GDS124_pairs > 0.013799
|   |   |   |   |   |   |   microarray_GDS124_pairs <= 0.044563: NO
```

```
|   |   |   |   |   |   |   microarray_GDS124_pairs > 0.044563: YES
|   |   |   |   |   yeastProteome_paralogs > 0.007397
|   |   |   |   |   |   microarray_GDS124_pairs <= 0.009801
|   |   |   |   |   |   |   yeast2hybrid <= 0.009812
|   |   |   |   |   |   |   |   microarray_GDS124_pairs <= 0.009508: NO
|   |   |   |   |   |   |   |   microarray_GDS124_pairs > 0.009508
|   |   |   |   |   |   |   |   |   microarray_GDS124_pairs <= 0.009584: YES
|   |   |   |   |   |   |   |   |   microarray_GDS124_pairs > 0.009584: NO
|   |   |   |   |   |   |   yeast2hybrid > 0.009812
|   |   |   |   |   |   |   |   microarray_GDS124_pairs <= 0.008145: NO
|   |   |   |   |   |   |   |   microarray_GDS124_pairs > 0.008145
|   |   |   |   |   |   |   |   |   microarray_GDS124_pairs <= 0.009008: YES
|   |   |   |   |   |   |   |   |   microarray_GDS124_pairs > 0.009008: NO
|   |   |   |   |   |   microarray_GDS124_pairs > 0.009801: YES
|   |   |   yeast2hybrid > 0.01
|   |   |   |   microarray_GDS124_pairs <= 0.010804
|   |   |   |   |   UCSFLocalization <= 0.007428
|   |   |   |   |   |   microarray_GDS124_pairs <= 0.009801: YES
|   |   |   |   |   |   microarray_GDS124_pairs > 0.009801
|   |   |   |   |   |   |   microarray_GDS124_pairs <= 0.01: NO
|   |   |   |   |   |   |   microarray_GDS124_pairs > 0.01
|   |   |   |   |   |   |   |   yeastProteome_paralogs <= 0.007397: YES
|   |   |   |   |   |   |   |   yeastProteome_paralogs > 0.007397: NO
|   |   |   |   |   UCSFLocalization > 0.007428: YES
|   |   |   |   microarray_GDS124_pairs > 0.010804: YES
|   |   yeastProteome_paralogs > 0.01: YES
```

C. YIR003W and YIL034C. YIR003W is predicted to be a component of the actin cortical patch or the F-actin capping protein complex, based in part on its predicted similarity to YIL034C. The process of predicting the CC similarity of these two proteins illustrates that the decision is based primarily on the fact that they have been observed to co-localize and to occur in the same complex experimentally. Note, however, that even when evidence is missing or negative it can influence the final network structure (as illustrated in the decision tree) and provide higher confidence in final predictions.

Stage 1 – Evidence (available at http://bmc-140.mit.edu/avid for all predictions)

UCSF localization

these proteins are reported to co-localize in the UCSF localization study, $P_{UCSF,CC}^{AVID1} = 0.058$

Yeast 2-hybrid

these proteins are included high-throughput yeast 2-hybrid, but are not observed to interact in any assay, $P_{Y2H,CC}^{AVID1} = 0.01$

MIPS complex

these proteins are part of a common complex described in MIPS, $P_{complex,CC}^{AVID1} = 0.263$

sequence similarity

there is not a significant sequence similarity between these proteins, $P_{seq,CC}^{AVID1} = 0.01$ (default)

mRNA profile

there is mRNA expression profile data for these two proteins, and the correlation coefficient for the two mRNA profiles (R = 0.8022) correlates fairly well with MF, $P_{mRNA,CC}^{AVID1} = 0.039$

Stage 2 - Filtering

P_{CC}^{AVID2} for this pair is the product of 100 times the correlation coefficients determined in Stage 1, with a value of 1.0 (= 100 x 0.01) used when there is no data.
P_{CC}^{AVID2} = 594.906 is greater than 12.8, so this pair passes the Stage 2 filter.

Stage 3 - The CC Decision Tree
The $P_{i,j}^{AVID1}$ values are fed to a decision tree, and are used as the basis for a series of binary decisions about whether two proteins are functional related (YES = related; NO = not related). The path through the tree for YIR003W and YIL034C is underlined. The same tree structure is used for all CC decisions.

```
UCSFLocalization <= 0.01
|   yeast2hybrid <= 0.009578
|   |   yeastProteome_paralogs <= 0.009608: NO
|   |   yeastProteome_paralogs > 0.009608
|   |   |   yeastProteome_paralogs <= 0.01
|   |   |   |   microarray_GDS124_pairs <= 0.010597: NO
|   |   |   |   microarray_GDS124_pairs > 0.010597
|   |   |   |   |   UCSFLocalization <= 0.003897: NO
|   |   |   |   |   UCSFLocalization > 0.003897: YES
|   |   |   yeastProteome_paralogs > 0.01
|   |   |   |   UCSFLocalization <= 0.003897: NO
|   |   |   |   UCSFLocalization > 0.003897
|   |   |   |   |   microarray_GDS124_pairs <= 0.016746
|   |   |   |   |   |   microarray_GDS124_pairs <= 0.01
|   |   |   |   |   |   |   microarray_GDS124_pairs <= 0.008134: YES
|   |   |   |   |   |   |   microarray_GDS124_pairs > 0.008134
|   |   |   |   |   |   |   |   microarray_GDS124_pairs <= 0.009059: NO
|   |   |   |   |   |   |   |   microarray_GDS124_pairs > 0.009059: YES
|   |   |   |   |   |   microarray_GDS124_pairs > 0.01
|   |   |   |   |   |   |   microarray_GDS124_pairs <= 0.011576: NO
|   |   |   |   |   |   |   microarray_GDS124_pairs > 0.011576
|   |   |   |   |   |   |   |   microarray_GDS124_pairs <= 0.015268: YES
|   |   |   |   |   |   |   |   microarray_GDS124_pairs > 0.015268: NO
|   |   |   |   |   microarray_GDS124_pairs > 0.016746: YES
|   yeast2hybrid > 0.009578
|   |   mips_complex <= 0.01
|   |   |   microarray_GDS124_pairs <= 0.015268
|   |   |   |   UCSFLocalization <= 0.003897: NO
|   |   |   |   UCSFLocalization > 0.003897
|   |   |   |   |   yeastProteome_paralogs <= 0.01
|   |   |   |   |   |   yeastProteome_paralogs <= 0.009608
|   |   |   |   |   |   |   microarray_GDS124_pairs <= 0.010597: NO
|   |   |   |   |   |   |   microarray_GDS124_pairs > 0.010597: YES
|   |   |   |   |   |   yeastProteome_paralogs > 0.009608: NO
|   |   |   |   |   yeastProteome_paralogs > 0.01
|   |   |   |   |   |   microarray_GDS124_pairs <= 0.010597
|   |   |   |   |   |   |   microarray_GDS124_pairs <= 0.00957
|   |   |   |   |   |   |   |   microarray_GDS124_pairs <= 0.007615: YES
|   |   |   |   |   |   |   |   microarray_GDS124_pairs > 0.007615: NO
|   |   |   |   |   |   |   microarray_GDS124_pairs > 0.00957: YES
|   |   |   |   |   |   microarray_GDS124_pairs > 0.010597: NO
|   |   |   microarray_GDS124_pairs > 0.015268
|   |   |   |   mips_complex <= 0.002335: NO
|   |   |   |   mips_complex > 0.002335: YES
|   |   mips_complex > 0.01
|   |   |   UCSFLocalization <= 0.003897
```

```
|   |   |   |   yeast2hybrid <= 0.01
|   |   |   |   |   yeastProteome_paralogs <= 0.01
|   |   |   |   |   |   yeastProteome_paralogs <= 0.009608
|   |   |   |   |   |   |   microarray_GDS124_pairs <= 0.016746: NO
|   |   |   |   |   |   |   microarray_GDS124_pairs > 0.016746
|   |   |   |   |   |   |   |   microarray_GDS124_pairs <= 0.026034: YES
|   |   |   |   |   |   |   |   microarray_GDS124_pairs > 0.026034
|   |   |   |   |   |   |   |   |   microarray_GDS124_pairs <= 0.039164: NO
|   |   |   |   |   |   |   |   |   microarray_GDS124_pairs > 0.039164: YES
|   |   |   |   |   |   yeastProteome_paralogs > 0.009608
|   |   |   |   |   |   |   microarray_GDS124_pairs <= 0.026034: NO
|   |   |   |   |   |   |   microarray_GDS124_pairs > 0.026034: YES
|   |   |   |   |   yeastProteome_paralogs > 0.01
|   |   |   |   |   |   microarray_GDS124_pairs <= 0.007572: NO
|   |   |   |   |   |   microarray_GDS124_pairs > 0.007572: YES
|   |   |   |   yeast2hybrid > 0.01
|   |   |   |   |   microarray_GDS124_pairs <= 0.008652: YES
|   |   |   |   |   microarray_GDS124_pairs > 0.008652
|   |   |   |   |   |   microarray_GDS124_pairs <= 0.011576
|   |   |   |   |   |   |   microarray_GDS124_pairs <= 0.01: NO
|   |   |   |   |   |   |   microarray_GDS124_pairs > 0.01
|   |   |   |   |   |   |   |   microarray_GDS124_pairs <= 0.010597: YES
|   |   |   |   |   |   |   |   microarray_GDS124_pairs > 0.010597
|   |   |   |   |   |   |   |   |   yeastProteome_paralogs <= 0.01: NO
|   |   |   |   |   |   |   |   |   yeastProteome_paralogs > 0.01: YES
|   |   |   |   |   |   microarray_GDS124_pairs > 0.011576: YES
|   |   |   UCSFLocalization > 0.003897: YES
UCSFLocalization > 0.01
|   mips_complex <= 0.01
|   |   microarray_GDS124_pairs <= 0.012112
|   |   |   mips_complex <= 0.002335
|   |   |   |   microarray_GDS124_pairs <= 0.007537: YES
|   |   |   |   microarray_GDS124_pairs > 0.007537: NO
|   |   |   mips_complex > 0.002335
|   |   |   |   microarray_GDS124_pairs <= 0.007496: NO
|   |   |   |   microarray_GDS124_pairs > 0.007496
|   |   |   |   |   microarray_GDS124_pairs <= 0.01
|   |   |   |   |   |   yeast2hybrid <= 0.009578
|   |   |   |   |   |   |   microarray_GDS124_pairs <= 0.00957: YES
|   |   |   |   |   |   |   microarray_GDS124_pairs > 0.00957: NO
|   |   |   |   |   |   yeast2hybrid > 0.009578
|   |   |   |   |   |   |   yeastProteome_paralogs <= 0.009608
|   |   |   |   |   |   |   |   microarray_GDS124_pairs <= 0.008134: NO
|   |   |   |   |   |   |   |   microarray_GDS124_pairs > 0.008134: YES
|   |   |   |   |   |   |   yeastProteome_paralogs > 0.009608: YES
|   |   |   |   |   microarray_GDS124_pairs > 0.01
|   |   |   |   |   |   yeast2hybrid <= 0.009578
|   |   |   |   |   |   |   microarray_GDS124_pairs <= 0.011576
|   |   |   |   |   |   |   |   microarray_GDS124_pairs <= 0.010597: NO
|   |   |   |   |   |   |   |   microarray_GDS124_pairs > 0.010597: YES
|   |   |   |   |   |   |   microarray_GDS124_pairs > 0.011576: NO
|   |   |   |   |   |   yeast2hybrid > 0.009578
|   |   |   |   |   |   |   microarray_GDS124_pairs <= 0.011576: NO
|   |   |   |   |   |   |   microarray_GDS124_pairs > 0.011576: YES
|   |   microarray_GDS124_pairs > 0.012112
|   |   |   yeastProteome_paralogs <= 0.01: NO
|   |   |   yeastProteome_paralogs > 0.01
|   |   |   |   microarray_GDS124_pairs <= 0.026034: NO
```

```
|   |   |   |   microarray_GDS124_pairs > 0.026034: YES
|   mips complex > 0.01: YES
```
