# Supplementary material for: AVID: An integrative framework for discovering functional relationships among proteins
Source: BMC Bioinformatics. 2005 Jun 1;6:136. doi: 10.1186/1471-2105-6-136 (PMC1177925; doi:10.1186/1471-2105-6-136)

Jiang & Keating

Additional file 3. Connectivity of networks used for testing (open circles) and final predictions (closed circles). The distribution of edges/node (connectivity) is plotted for the molecular function, biological process and cellular component networks.

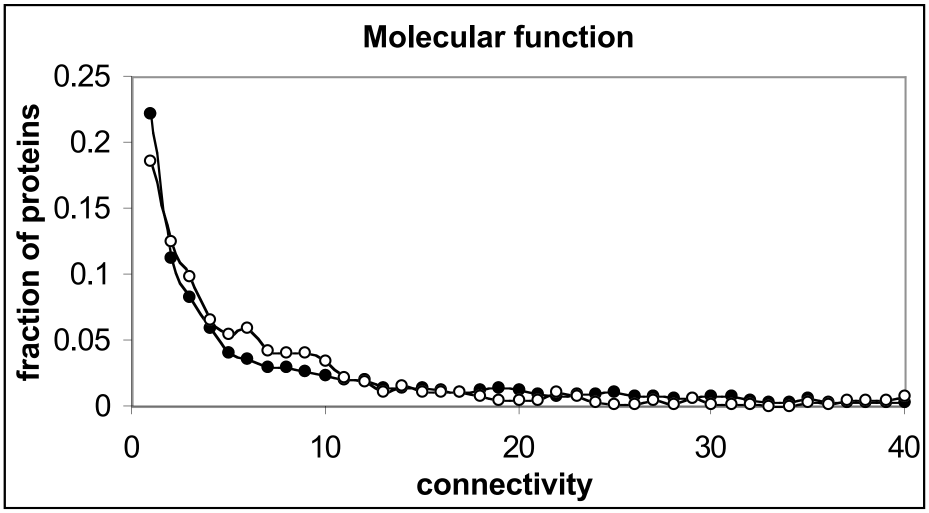

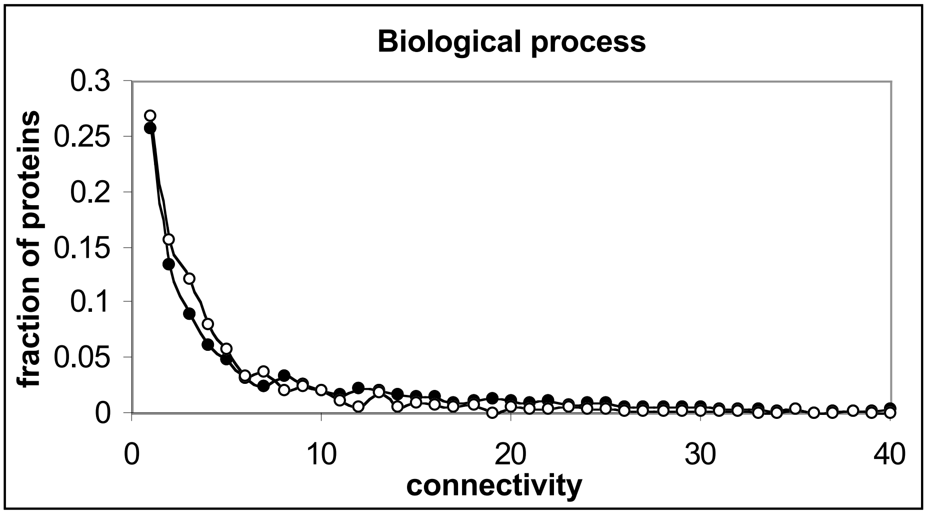

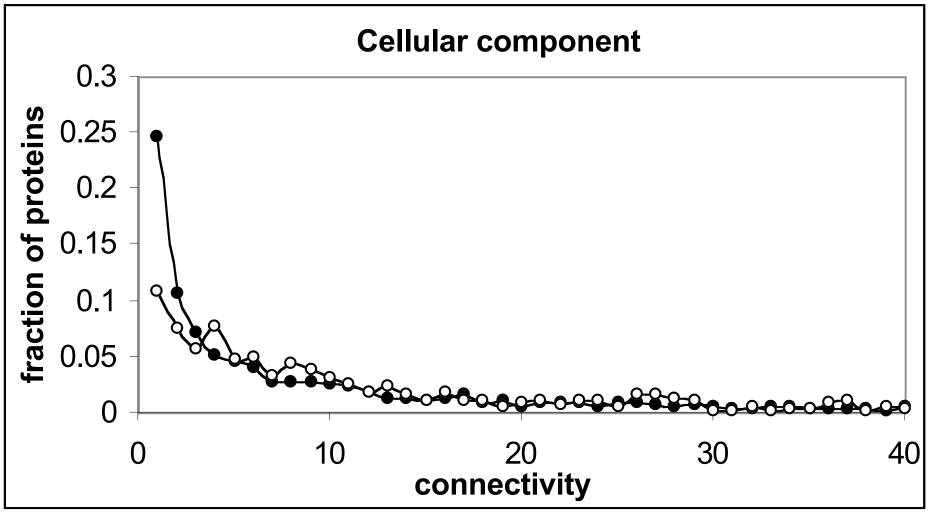

Supplement: Additional File 3 — Connectivity plots comparing the testing and prediction networks. [file 1471-2105-6-136-S3.pdf]
